# Development of Slow-Release Salt Storage Fillers and Performance Evaluation of Salt-Storage Pavement

**DOI:** 10.3390/ma19122450

**Published:** 2026-06-08

**Authors:** Yanhai Yang, Dongning Ban, Ye Yang, Guanliang Chen

**Affiliations:** 1 School of Transportation and Geomatics Engineering, Shenyang Jianzhu University, Shenyang 110168, China; yangyanhai168@126.com (Y.Y.); 15940086799@163.com (D.B.); 2 Guanliang Chen School of Civil and Environmental Engineering, Southwest Jiaotong University, Chengdu 610031, China; chenguanliang@my.swjtu.edu.cn

**Keywords:** slow-release salt-storage filler, salt-storage asphalt mixture, road performance, epoxy resin coating, snow-melting and ice-suppressing

## Abstract

To address the issues of poor sustained-release behavior and limited long-term efficacy associated with conventional salt-storage materials, this study developed the epoxy-resin-encapsulated slow-release salt-storage filler to enhance both the engineering performance and the deicing/snow-melting capacity of salt-storage pavements. In this study, attapulgite was optimized and selected as the salt storage carrier through the adoption of pesticide coating technology and experimental testing, wherein a deicing salt blend with a CaCl_2_ to NaCl mass ratio of 2:1 was loaded via a wet adsorption method. Subsequently, using dimethicone as the surface modifier, the optimal encapsulation process was determined to involve the dilution ratio of epoxy resin to cyclohexanone of 4:1 and the curing agent dosage of 30% by weight. The results indicated that the recommended content of the filler should not exceed 5%. The filler reduced the high-temperature stability and water stability of the mixture, while the low-temperature crack resistance first increased and then decreased, peaking at the 2% filler content with an improvement of 12.2%. The water stability was the most significantly affected by the filler content. Ice–snow melting performance tests demonstrated that the salt-storage mixture with 5% filler achieved the deicing rate of 56.35% at −5 °C, meeting the industry standard requirements. The self-prepared slow-release salt-storage filler exhibited superior long-term ice–snow melting performance to V-260, with the slow-release duration extended by 60%. The salt release process was divided into three distinct stages: rapid dissolution, stable release and slow dissolution. The 60 °C was determined as the optimal temperature for the accelerated immersion testing, which the accelerated test could effectively simulate the natural immersion process. Based on the prediction model established accordingly, the functional service life of snow-melting for this slow-release salt-storage asphalt pavement in northern area was estimated be approximately 4.07 years. The slow-release salt-storage filler fabricated in this work possesses both remarkable sustained-release behavior and deicing efficacy. The findings provide the technical foundation for the development of novel salt-storage pavement materials, performance characterization, and mechanistic analysis of snow-ice melting.

## 1. Introduction

The rapid expansion of highway transportation infrastructure has become the cornerstone of modern economic development. By the end of 2024, the total road mileage in China had reached 5.49 million kilometers, underscoring the critical role of road networks in societal mobility and economic activity [1]. However, winter weather conditions, particularly snow accumulation and pavement icing, pose severe threats to traffic safety and operational efficiency. The presence of ice and snow significantly degrades pavement skid resistance. While the friction coefficient of dry asphalt pavement is approximately 0.6, it drops precipitously to 0.2 under snow cover and to as low as 0.15 on icy surfaces—representing reductions of 66.7% and 75.0%, respectively [1]. This loss of surface friction leads to vehicle skidding, extended braking distances, and a heightened risk of accidents, thereby disrupting normal traffic flow and endangering both life and property. Consequently, the development of effective and sustainable pavement deicing and anti-icing technologies remains a persistent challenge for transportation agencies worldwide.

Current strategies for snow and ice removal are broadly categorized into “passive” and “active” methods. Passive methods encompass manual clearing, mechanical removal, and the application of chemical deicers or abrasives [2]. While manual and mechanical methods are widely practiced, they are often labor-intensive, can delay traffic restoration, and may induce physical damage to pavement surfaces and markings [3,4,5]. The application of chloride-based salts remains the most prevalent chemical approach due to its low cost and high efficacy [6]. Nevertheless, the environmental drawbacks of chloride salts—including corrosion of steel infrastructure, degradation of roadside vegetation, and contamination of soil and water sources—are well documented and increasingly unsustainable [7]. Active methods, such as thermal heating and conductive concrete, offer high deicing efficiency but involve substantial capital investment, complex installation, and high energy consumption, limiting their widespread application [8,9,10]. Other active strategies, including superelastic rubber particle pavements that utilize mechanical deformation to break ice bonds, have shown promise but may require complementary treatments for complete ice clearance [11,12,13,14].

In response to the limitations of conventional passive and active approaches, salt-storage pavement technology has emerged as a promising functional pavement solution with intrinsic anti-icing and deicing capabilities [1,15]. This technology incorporates salt-storage fillers—materials designed to release freezing-point depressants gradually—into the asphalt mixture. Under the combined action of traffic loading and moisture infiltration, these fillers slowly release active ions that migrate to the pavement surface, lowering the freezing point of surface water and weakening the adhesive bond between ice and pavement [2,16]. The concept of incorporating deicing chemicals into pavement materials dates back to the 1960s in Switzerland with the development of Verglimit, and subsequent innovations in Japan produced MFL, the widely used salt-storage filler capable of maintaining low ice adhesion for 6–10 years [17,18]. Over the decades, various salt-storage material formulations have been investigated, including surface-oiled granules, cementitious encapsulated aggregates, and powdered porous carriers impregnated with salts [19,20,21,22]. In China, research efforts have yielded proprietary products such as Icebane and emulsified asphalt coatings incorporating anti-freezing agents, aiming to overcome the performance and cost limitations of imported technologies [17,23,24].

Despite the considerable progress in salt-storage pavement technology, several critical challenges persist. Foremost among these is the issue of release durability. Conventional salt-storage fillers often exhibit rapid initial salt elution, leading to a short functional lifespan and diminishing long-term deicing performance. This burst release not only reduces the pavement’s long-term efficacy but may also cause moisture absorption on the pavement surface, potentially compromising skid resistance and accelerating moisture-induced pavement distress [25]. Furthermore, the incorporation of hygroscopic salts has been associated with concerns regarding moisture susceptibility, reduced fatigue life, and complications in asphalt mixture recycling [18,26,27]. Although research has elucidated the macroscopic mechanisms of ice melting—namely, vapor pressure depression, freezing point lowering governed by Raoult’s law, and the formation of the lubricating brine film at the ice–pavement interface [3,28,29,30]—there remains the need for a more profound understanding of the micro-scale transport phenomena and diffusion kinetics that govern salt migration within the porous asphalt matrix [31].

To address the poor slow-release performance and lack of long-term effectiveness commonly observed in salt-storage materials, in this study, the prepared snow-melting material was subjected to encapsulation treatment using epoxy resin to obtain the slow-release salt-storage material. This treatment endows the material with sustained-release capability, thereby enhancing the service efficiency and prolonging the service life of salt-storage asphalt pavement.

## 2. Experimental Section

### 2.1. Preparation of Sustained-Release Salt-Retaining Mixture

Based on the bulk density of various aggregates and the mix ratio, the optimal data for the AC-13 type was estimated to be 5.0%. By conducting tests on 5 types of specimens, the basic data, such as the bulk density, were measured, and the test data results are shown in Table 1.

When calculating the values relative to the maximum density a_1_, the maximum stability a_2_, the median porosity a_3_, and the median asphalt saturation a_4_, all match the range of the asphalt-sand ratio. Among them, the optimal data is calculated based on Equations (1)–(3).

(1) $$OAC_{1}=(a_{1}+a_{2}+a_{3}+a_{4})/4$$

(2) $$OAC_{2}=(OAC_{min}+OAC_{max})/2$$

(3) $$OAC=(OAC_{1}+OAC_{2})/2$$

where OAC_1_ refers to the initial value used in the calculation formula introduced earlier to represent the optimal asphalt-aggregate ratio. OAC_2_ refers to the median value used in the calculation formula introduced earlier to represent the standard asphalt-aggregate ratio when all indicators match. OAC_min_ refers to the minimum value of the asphalt-aggregate ratio in the calculation formula introduced earlier. OAC_max_ refers to the maximum value of the asphalt-aggregate ratio in the calculation formula introduced earlier.

When calculating the median amount of asphalt used in this paper, a specific average value data was set as OAC_1_ = 5.0%. By using the median of the dosage range OAC_min_ to OAC_max_, which matches all aspects to the standard, OAC_2_ = 4.85% was obtained. Finally, the median of OAC_1_ and OAC_2_ was taken as the corresponding optimal asphalt-aggregate ratio (OAC), and the optimal data under this synthetic gradation was selected as 4.9%.

### 2.2. Experimental Method

#### 2.2.1. Wheel Tracking Test

The 300 mm × 300 mm × 50 mm plate-shaped specimen was formed using a rutting forming instrument. The polymer-modified asphalt mixture was placed at room temperature for 48 h as appropriate [32,33]. Usually, the deformation at 45 min and 60 min was taken, and the number of times the test wheel traveled back and forth to produce 1 mm deformation was calculated as the dynamic stability. This was calculated according to Equation (4). Rutting tests were conducted on AC-13 rutting specimens with different salt storage filler contents to analyze the influence of the content on stability.

(4) $$DS=\frac{t_{2}-t_{1}}{d_{2}-d_{1}}\times C_{1}$$

where DS denotes Dynamic Stability of Asphalt Mixture, cycles/mm; d_2_ denotes Deformation of the specimen at a test duration of 60 min, mm; d_1_ denotes Deformation of the specimen at a test duration of 45 min, mm; C_1_ denotes Specimen Width Factor.

#### 2.2.2. Low-Temperature Bending Test

Slab specimens with dimensions of 300 mm × 300 mm × 50 mm were molded by the rutting tester, after which mixture beams of 250 mm × 30 mm × 35 mm were cut along the rolling direction using the rock cutting machine. The specimens were then incubated in the low-temperature environment of −10 °C for no less than 45 min, with the spacing between any two specimens kept at no less than 10 mm during incubation.

During the test, the top and bottom orientation of the beams was kept consistent with that in the molding process. The beam span was set at 200 mm, and the test temperature was maintained at −10 °C. The concentrated load was applied to the beams at a loading rate of 50 mm/min until the specimens fractured and failed. Indicators such as the flexural failure strength and failure flexural-tensile strain of the beams were measured, and the maximum flexural-tensile strain was selected as the evaluation index. The larger the maximum flexural-tensile strain, the better the low-temperature cracking resistance of the asphalt mixture [34].

#### 2.2.3. Immersion Marshall Test

Standard Marshall specimens were placed in a constant-temperature water bath preheated to 60 °C ± 1 °C for heat preservation, with the holding time ranging from 30 to 40 min. A certain interval should be kept between the specimens, which should be padded up and kept at a distance of no less than 5 cm from the bottom of the water bath. The difference between the immersion Marshall test and the standard Marshall test is that the specimens are incubated in the 60 °C constant-temperature water bath for 48 h [35]. After that, the cured specimens were placed on the Marshall tester (Beijing Xingke Instrument Co., Beijing, China) for testing, and the loading rate should be maintained at 50 mm/min ± 5 mm/min.

#### 2.2.4. Freeze–Thaw Splitting Test

The mixture containing salt-storage filler was divided into two groups. One group was immersed in the water bath at 25 °C for 2 consecutive hours, after which the specific tensile strength, denoted as R_T1_, was measured [36]. Subsequently, the specimens were subjected to vacuum pumping at a pressure of 0.09 MPa for 15 min. After restoring to normal pressure, they were left to stand for 0.5 h, then placed at −18 °C for 16 h, followed by heat preservation at a constant temperature of 60 °C for 24 h. After that, the specimens were immersed again in a 25 °C water bath for 2 consecutive hours, and the specific tensile strength, denoted as R_T2_, was measured. The specific strength ratio, TSR, was calculated using the formula: TSR = (R_T2_/R_T1_) × 100%. The specific tensile strength was determined in accordance with Formula (5).

(5) $$R_{T}=\frac{0.006287P_{T}}{h}$$

where R_T_ rdenotes Splitting tensile strength, MPa denotes Maximum load at specimen failure, and N denotes Height of the specimen.

## 3. Preparation Method of Salt-Storage Filler

### 3.1. Molecular Simulation Results

Salt-storage materials are mainly prepared by incorporating a certain amount of snow-melting components into the pore structure of porous carriers. Therefore, their adsorption performance is derived from the porous carriers. The quality of the carriers will greatly affect the adsorption capacity, the actual dissipation rate, and the actual salt slow-release performance. Different types of carriers have varying adsorption capacities, which are influenced by their structural characteristics. The specific surface area of the carrier determines its adsorption capacity. As the specific surface area of the carrier increases, its capacity to adsorb soluble salts increases correspondingly, resulting in the higher salt content in the prepared materials and the relatively longer service life. Therefore, this study conducted BET specific surface area measurement and research on five types of carriers, namely fly ash, calcium carbonate, diatomite, attapulgite, and volcanic rock. The obtained numerical results are shown in Table 2.

**Figure 1:**
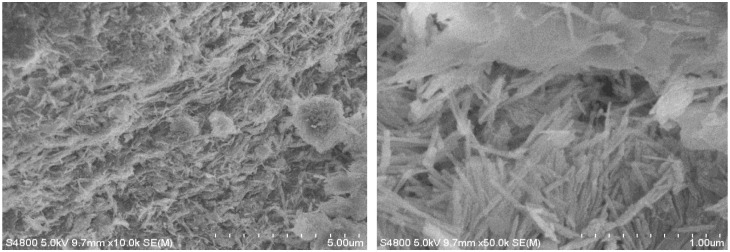
Attapulgite.

The adsorption performance of attapulgite is affected by a variety of factors, including its physical and chemical properties; characteristics such as the high specific surface area also exert an influence on its adsorption capacity. The special feature of attapulgite lies in its layer-chain structure, which presents the needle-like or fibrous morphology. As shown in Figure 1, observations under a scanning electron microscope (SEM: KYKY Technology Co., Beijing, China) confirm that attapulgite has a needle-like or fibrous morphology. This morphology contributes to its excellent adsorption performance, which is why attapulgite clay is selected as the carrier.

### 3.2. Selection of Adsorption Methods

**Figure 2:**
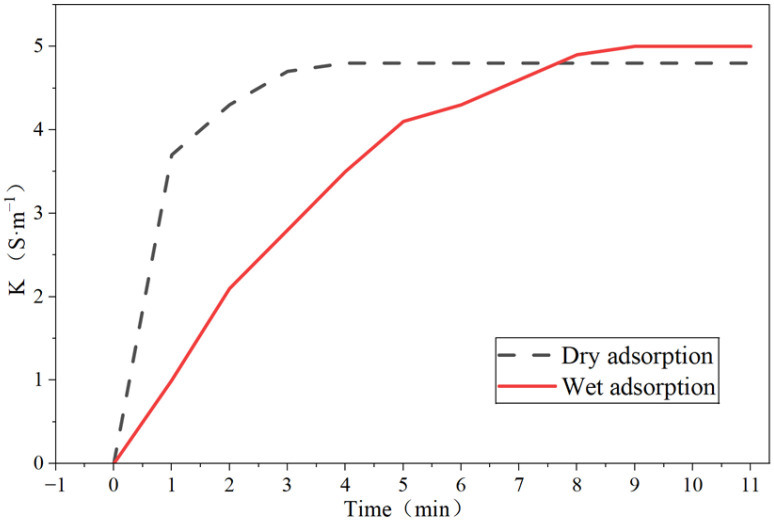
Variation in conductivity under different preparation methods.

Salt storage materials were prepared by two methods, namely wet adsorption and dry adsorption. Specifically, 36 g of sodium chloride (NaCl) and 20 g of the carrier were weighed out separately for the preparation process. The dissolution rates of the salt storage materials fabricated via the two methods were obtained through conductivity tests, and the adsorption capacity of the carrier for NaCl was also determined. The results showed that the higher the adsorption capacity of the carrier for NaCl, the slower the dissolution rate; conversely, the lower the adsorption capacity, the faster the dissolution rate. The changes in the dissolution conductivity of the salt contained in the salt storage materials prepared by the two adsorption methods are illustrated in Figure 2.

After the salt components in the salt storage materials prepared by the wet and dry methods were fully dissolved out, the obtained conductivity values were 4.97 K/S·m^−1^ and 4.81 K/S·m^−1^, respectively, indicating that the adsorption capacity of the wet method was slightly higher than that of the dry method.

Meanwhile, in terms of dissolution rate comparison, the conductivity of the material prepared by the dry adsorption method reached the peak in 4 min and increased nearly linearly; whereas the material obtained by the wet adsorption method did not reach the limit until 8 min, at which point the NaCl was completely dissolved.

This proves that the material prepared by the wet adsorption method has a more sufficient adsorption of salt components and a better dissolution effect than that prepared by the dry method. Therefore, the wet adsorption method is selected as the preparation process for the production of salt storage materials.

### 3.3. Selection of Modifiers

The nonionic surfactant Span-60 is synthesized by the esterification of glycerol and monostearic acid. Thanks to the ester bonds between the acidic monostearate groups and alcoholic glycerol groups in its molecular structure, it forms tiny micellar structures in water, thereby reducing the surface tension of the liquid and enabling liquid molecules to disperse and mix with each other more easily.

The organosilicon water repellent SHP-60 can form a continuous and dense siloxane layer on the surface of materials, thus enhancing the surface hydrophobicity of the materials. Dimethyl silicone oil is a class of important polymeric organosilicon materials. It can form the siloxane layer to reduce surface energy and enhance surface hydrophobicity.

**Figure 3:**
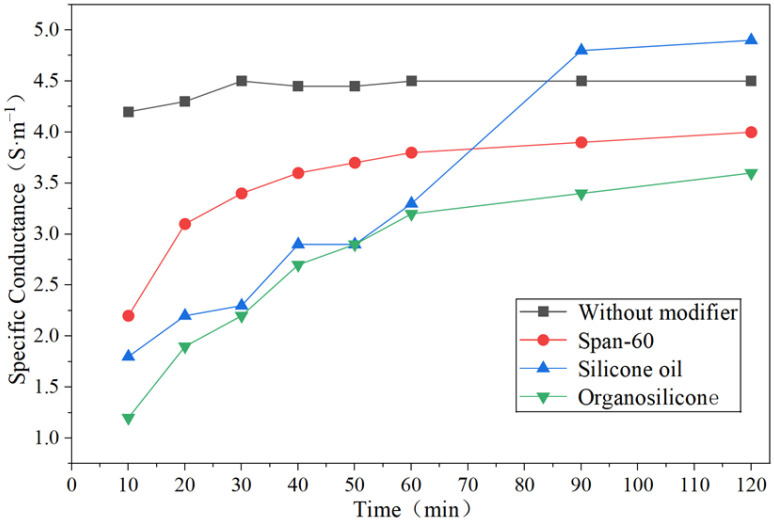
Comparison of modification effects of different water repellents.

To verify the effect of hydrophobic treatment, the conductivity of the salt storage carrier was measured within 2 h and compared with that of the untreated sample. The test results are shown in Figure 3.

Span-60 exhibits the poorest slow-release performance; the release is completed at 60 min, and its slow-release effect in the early stage is also inferior to that of the other two materials. Dimethyl silicone oil and organosilicon show almost identical slow-release effects in the early stage. However, from the perspective of release rate in the later stage, although dimethyl silicone oil has a slightly faster release rate than organosilicon, the total salt content of the salt storage carrier treated with dimethyl silicone oil is higher than that treated with organosilicon.

From the operational perspective, the use of Span-60 requires a large dosage and complex operations, with strict temperature requirements. Moreover, absolute ethanol must be used as the solvent, which undoubtedly increases the cost and potential hazards of the experiment. Compared with dimethyl silicone oil, the organosilicon water repellent SHP-60 has stricter requirements for the stirring process, and it needs to be dried and then ground into powder. In contrast, dimethyl silicone oil not only achieves a better slow-release effect but also has fewer requirements for the stirring process, without being restricted by factors such as temperature and time.

In addition, after treating the salt storage carrier with dimethyl silicone oil, there is no need for grinding after drying, which is consistent with the state of the untreated carrier. Therefore, considering both the slow-release effect and operational feasibility, dimethyl silicone oil is the optimal choice.

### 3.4. Epoxy Resin Coating

#### 3.4.1. Determination of Dilution Ratio

**Figure 4:**
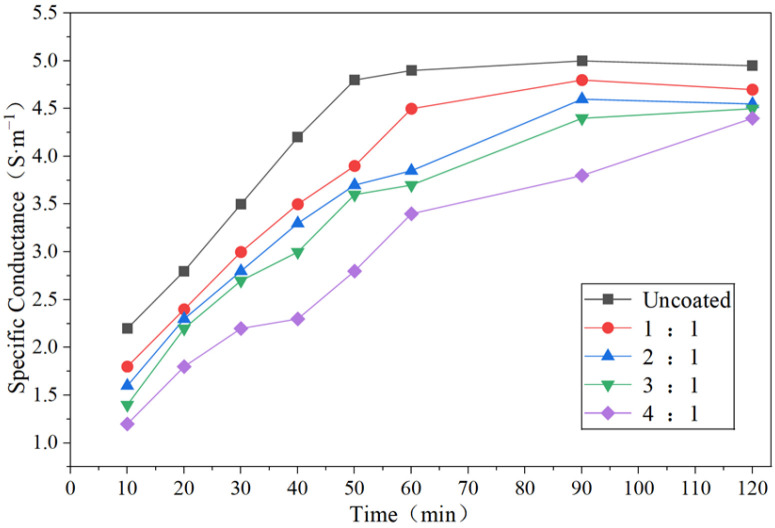
Effect of epoxy resin dilution ratio on slow-release performance.

Cyclohexanone was selected as the diluent for epoxy resin in this study due to its relatively low cost, which can accelerate the curing rate of epoxy resin. Epoxy resin-cyclohexanone solutions with different ratios were prepared, followed by the addition of 30% epoxy resin curing agent. After thorough stirring, the salt storage materials were immersed in the solutions with different dilution ratios. Once evenly soaked, the materials were taken out and placed in the oven (Shanghai Jingke Scientific Instrument Co., Shanghai, China) for curing at 100 °C. After curing, their conductivity was tested and compared with that of the uncoated salt storage materials. The test results are shown in Figure 4.

The slow-release performance of salt-storage materials can be improved after surface coating. Moreover, the actual slow-release performance tends to deteriorate with the increase in dilution ratio. The optimal slow-release effect is achieved at the dilution ratio of 4:1, with the release amount still increasing at 120 min. On the one hand, the material exhibits excellent slow-release behavior in the early stage; on the other hand, the salt-storage material can still maintain the sustained release even after other salts have been released at 90 min. With the continuous increase in dilution ratio, the coating solution becomes more diluted, leading to a thinner adsorbed coating layer. After curing, the formed coating film turns out to be thinner, which makes it easier for water to penetrate.

The release process consists of three distinct stages. First, water penetrates the salt crystals, leading to the gradual increase in the internal pressure of the particles; when the internal pressure exceeds the bearing capacity of the coating layer, the coating ruptures, resulting in the release of various salts, which is referred to as the “fracture mechanism”. If the coating can withstand the internal pressure, the salts will diffuse outward, driven by relevant forces, which is called the diffusion mechanism and is reflected by the continuous precipitation of chloride ions within the first 30 min. Second is the stable release stage, where the release follows a linear relationship, as evidenced by the slow and steady precipitation of chloride ions between approximately 30 min and 120 min. Third is the attenuation stage, during which the salt release rate decreases continuously until the conductivity value stabilizes at a constant level, indicating the completion of release after 120 min.

#### 3.4.2. Determination of Curing Agent Dosage

**Figure 5:**
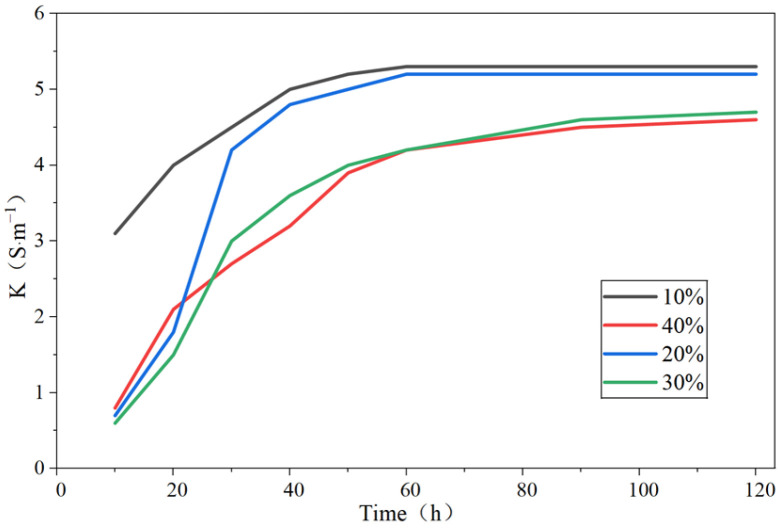
Effect of curing agent ratio on slow-release performance.

Diluted solutions with an epoxy resin-to-cyclohexanone mass ratio of 4:1 were prepared, and curing agents were added at dosages of 10%, 20%, 30%, and 40%, respectively. After thorough stirring, the salt storage materials were immersed in the various diluted solutions. Once the surfaces were fully soaked, the samples were cured at 100 °C. Upon completion of the experiment, conductivity tests were conducted for data measurement, and the obtained results are shown in Figure 5.

According to the results shown in Figure 5, the formulation with 30% curing agent dosage exhibits the optimal performance in the formation of primary amines, secondary amines, and tertiary amines. The reaction mechanism between the curing agent and epoxy resin is realized through ring-opening polymerization. The active groups (e.g., amines, acid anhydrides, etc.) in the curing agent react with the epoxy groups in the epoxy resin molecules to form ring-opened structures. These ring-opened structures then react with each other to form cross-linked structures, thus producing a stable solid material. Specifically, primary amine groups first react to form secondary amine groups, which then further react with epoxy groups to generate tertiary amine groups. Meanwhile, an etherification reaction occurs between hydroxyl groups and epoxy groups, ultimately forming the network structure [36]. Therefore, the 30% dosage condition was selected for the experiment. It should be noted that an insufficient dosage of curing agent may result in an incomplete curing reaction, which would fail to form the dense coating film. Thus, the optimal addition amount of epoxy resin curing agent was determined to be 30%.

In summary, the preparation of the slow-release salt-storage filler was carried out under the following optimal conditions: attapulgite was selected as the salt-storage carrier; the snow-melting salt was formulated by mixing calcium chloride (CaCl_2_) and sodium chloride (NaCl) at the mass ratio of 2:1; dimethyl silicone oil was adopted as the surface modifier. The salt-storage material was subjected to epoxy resin coating: the epoxy resin was diluted with cyclohexanone at a mass ratio of 4:1, and 30% of curing agent was added to the diluted solution. The salt-storage material was immersed in the prepared coating solution and then placed in the rotating drum to ensure uniform coating. After curing at 100 °C, the resulting slow-release salt-storage filler achieved the optimal slow-release performance.

## 4. Pavement Performance of Slow-Release Salt-Storage Mixtures with Different Dosages

### 4.1. High-Temperature Stability

**Figure 6:**
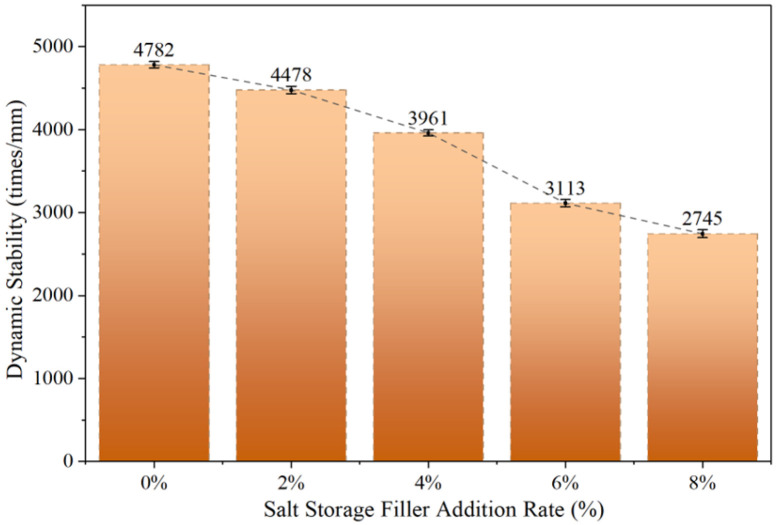
Effect of salt-storage filler content on high-temperature stability.

The dynamic stability of asphalt mixtures with different dosages of salt-storage fillers is shown in Figure 6. With the increase in the dosage of salt-storage fillers, the dynamic stability of the asphalt mixture rutting specimens decreases continuously. When the dosage increases from 0% to 4% and 6%, the dynamic stability decreases from 4782 times/mm to 3961 times/mm and 3113 times/mm, representing the reduction of 17.3% and 34.9%, respectively. Moreover, a significant decrease in dynamic stability is observed when the dosage rises from 4% to 6%.

With the increase in the dosage of salt-storage fillers, the high-temperature stability of asphalt mixtures decreases significantly. In salt-storage asphalt mixtures, the main reason for the decline in high-temperature stability is that the dissolution of salt components from the salt-storage materials impairs the adhesion between asphalt and mineral aggregates. Insufficient adhesion between asphalt and mineral aggregates will cause the mixture to decompose at high temperatures. Due to the mixing of salt particles and mineral materials, a special powdery material is formed. The salt components will reduce the interaction with asphalt, thus failing to form the relatively developed adsorption-dissolution film [37]. In addition, as the replacement ratio of salt-storage fillers increases, the content of structural asphalt in the salt-storage asphalt mortar increases while the content of free asphalt decreases. This makes the asphalt mortar harden; its cohesion decreases accordingly, and its shear strength also reduces.

### 4.2. Low-Temperature Stability

**Figure 7:**
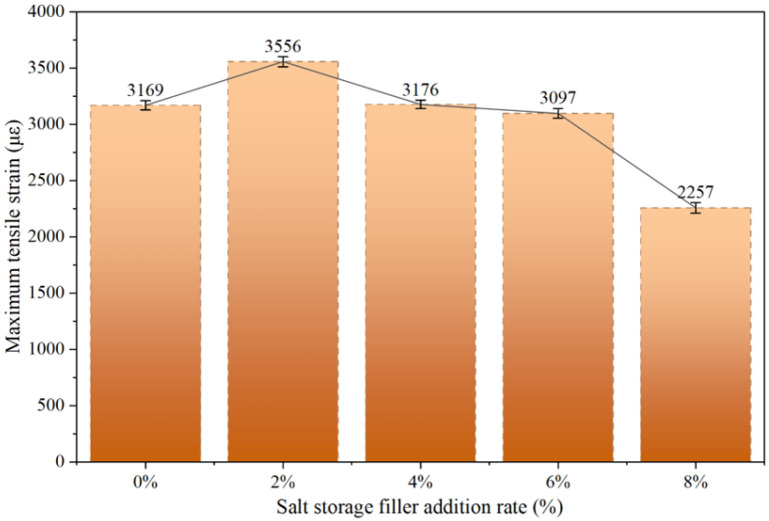
Effect of salt-storage filler content on low-temperature crack resistance.

The maximum flexural-tensile strain of asphalt mixtures with different salt-storage fillers at −10 °C is shown in Figure 7. The maximum flexural-tensile strain first increases and then decreases with the rise in filler dosage, reaching its peak when the dosage is 2%, with an increase of 12.2% in the maximum flexural-tensile strain. Moreover, when the filler dosage ranges from 0% to 4%, the maximum flexural-tensile strain is significantly higher than that of the asphalt mixture without any salt-storage filler added.

First, under low-temperature curing conditions, asphalt mixtures undergo physical hardening. Prolonged exposure to low temperatures leads to molecular aggregation and a certain reduction in molecular mobility, ultimately resulting in the deterioration of low-temperature performance [38]. Moreover, asphalt mixtures can be regarded as elastic materials, and their failure process is a process of energy dissipation. Work can be converted into the following forms of energy, which are then stored as corresponding elastic strain energy. Force transmission is achieved through mineral aggregate particles. After the actual occurrence of cracks, the propagation and diffusion of cracks mainly depend on the mixture itself. During crack propagation, the increase in fine-graded particles hinders the connection of microcracks and macrocracks, leading to longer crack propagation distances and greater energy consumption, thus resulting in an increase in flexural-tensile strength. However, with the increase in the dosage of salt-storage fillers, the total surface area of the fillers gradually increases, and the content of structural asphalt increases accordingly, while the actual content of free asphalt decreases [39].

Under low-temperature conditions, the asphalt mixture undergoes physical hardening, during which molecular aggregation reduces the degrees of freedom and consequently drives the material toward more elastic behavior. Concurrently, the increase in the content of salt-storage filler enlarges the total specific surface area of the filler, leading to a higher proportion of structural asphalt and the corresponding decrease in free asphalt. This shift results in diminished viscoelasticity and enhanced brittleness, which constitute the primary mechanisms governing the changes in elasticity and brittleness of the mixture.

### 4.3. Water Stability

**Figure 8:**
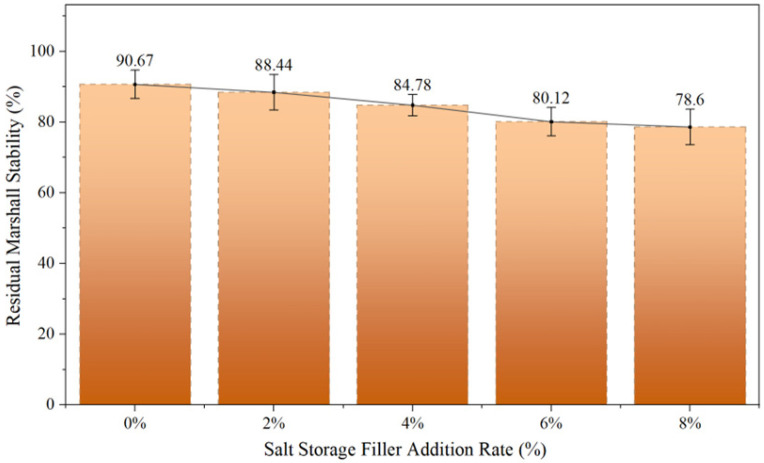
Results of immersion Marshall test at different dosages.

The residual Marshall stability of asphalt mixtures with different dosages of salt-storage fillers is shown in Figure 8. Compared with ordinary AC-13 asphalt mixtures, the immersion Marshall residual stability of salt-storage asphalt mixtures decreases with the increase in the dosage of salt-storage fillers. Specifically, the residual Marshall stability decreases by 2.5%, 6.5%, 11.6%, and 13.3% when the dosages of salt-storage fillers are 2%, 4%, 6% and 8%, respectively.

On the one hand, during the mixing process of the mixture, chloride ions (Cl^−^) and other components in the salt-storage filler will impair the adhesion between asphalt and aggregates, leading to the formation of unstable asphalt adsorption films on the aggregate surface, thus affecting the mixing quality of the mixture. On the other hand, when the mixture is exposed to a humid environment, water will penetrate into the interior of the salt-storage asphalt mixture through open pores. The salt-storage filler will dissolve due to water erosion, and porous structures will form in the asphalt mortar and the asphalt surface layer of aggregates, resulting in the stripping of the asphalt film [40]. Meanwhile, the coupling effect of water temperature in the immersion Marshall test will accelerate the release of salt. Combined with the influence of salt-storage filler on the asphalt adhesion performance, the higher the dosage of salt-storage filler, the greater the proportion of salt dissolution and segregation. This causes the structural asphalt inside the asphalt mixture to transform into free asphalt, leading to poor adhesion between asphalt and aggregates. Consequently, the internal structure of the salt-storage asphalt mixture becomes loose and the porosity increases, resulting in a serious decline in the water stability of the mixture [41].

**Figure 9:**
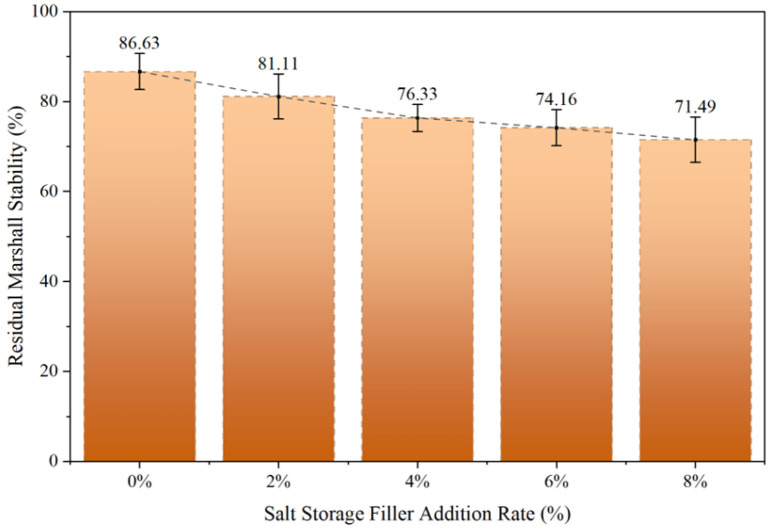
Freeze–thaw splitting test results of salt-storage asphalt mixtures with different dosages.

The freeze–thaw splitting residual strength ratio of salt-storage asphalt mixtures with different dosages is shown in Figure 9. Compared with ordinary AC-13 asphalt mixtures, the freeze–thaw splitting strength of salt-storage asphalt mixtures also decreases with the increase in the dosage of salt-storage fillers. This test result is consistent with that of the immersion Marshall test, but with the relatively large dispersion.

The salt-storage asphalt mixture contains salt-storage fillers, which undergo deliquescence at the filler locations. This process significantly increases the number of pores and porosity, thereby expanding the contact area with water [42]. Meanwhile, freeze–thaw cycles impair the performance of asphalt mortar and reduce its interfacial bonding strength. As damage accumulates, microcracks initiate, propagate, overlap, and connect. This ultimately leads to an increase in the voids of the mixture at the mesoscopic level, which raises the size and quantity of pores. These changes accelerate the dissolution and segregation of salt, thereby exacerbating freeze–thaw damage and resulting in the decrease in the freeze–thaw resistance of the asphalt mixture [43,44]. Therefore, the increase in the content of salt-storage fillers will reduce the actual water stability of the mixture.

### 4.4. Integrated Discussion

The salt-storage filler exhibits a dual-role behavior within the asphalt mixture matrix, functioning simultaneously as a reinforcing particulate phase and a potential degradation source through salt dissolution. This intrinsic mechanistic duality governs the observed evolution of pavement performance properties in a unified manner. At low incorporation levels (≤2% by mass), the physical filler effect predominates: the discrete filler particles are homogeneously dispersed within the asphalt mastic, increasing the specific surface area of the fine aggregate fraction and promoting the formation of structured asphalt films. Concurrently, the presence of fine-grade particles impedes microcrack propagation by extending crack trajectories and enhancing energy dissipation capacity, manifesting as a significant improvement in low-temperature crack resistance (12.2% increase in maximum flexural-tensile strain at 2% dosage). However, as the filler dosage increases beyond this threshold (>2%), the salt dissolution-induced degradation effect progressively intensifies and becomes dominant: the leaching of saline components from the filler compromises the adhesive bonding between asphalt binder and mineral aggregates, resulting in the deficient development of adsorption–dissolution interfacial films; simultaneously, the proportion of structured asphalt increases abnormally at the expense of free asphalt, leading to mastic embrittlement and reduced cohesive strength. This mechanistic transition consistently explains the following performance trends:

(1) Monotonic deterioration of high-temperature stability: the interfacial debonding caused by salt dissolution facilitates aggregate slippage and structural disintegration under elevated temperatures, resulting in a continuous decline in dynamic stability (34.9% reduction from 0% to 6% dosage);

(2) Progressive degradation of moisture damage resistance: the dissolution of salt creates porous structures within the mastic and at aggregate surfaces upon water ingress, accelerating asphalt film stripping; coupled with the water–temperature synergistic effect that further promotes salt elution, a self-amplifying “salt-leaching–porosity–moisture damage” cycle is established, causing consistent reductions in both immersion Marshall residual stability and freeze–thaw splitting strength ratio;

(3) Non-monotonic evolution of low-temperature performance: the physical filler reinforcement and crack-arresting mechanisms prevail at low dosages, whereas mastic embrittlement and reduced viscoelasticity dominate at higher dosages, producing the observed peak in flexural-tensile strain at 2% dosage followed by a subsequent decline.

In summary, moisture damage resistance exhibits the highest sensitivity to salt-storage filler content, as it is subject to the compounded amplification of both direct salt erosion and porosity enhancement. It is recommended that the filler dosage be limited to 5% by mass to ensure adequate snow-melting functionality while constraining the dissolution-induced degradation effects within acceptable thresholds.

## 5. Analysis on Ice and Snow Melting Performance of Slow-Release Salt-Storage Asphalt Mixture

### 5.1. Test on Snow Melting and Ice Suppression Performance of Slow-Release Salt-Storage Asphalt Mixture

#### 5.1.1. Test on Short-Term Ice and Snow Melting Performance

The snow melting performance was tested using the snow melting method. Using rutting plates, the tests were conducted under natural or artificial snowfall conditions, with ambient temperature and snowfall amount recorded. A qualitative analysis was then carried out by directly comparing the snow accumulation on the rutting plates. Salt-storage fillers were incorporated into the asphalt mixture at a dosage of 5% to prepare salt-storage asphalt mixture rutting specimens. These specimens were compared with ordinary AC-13 rutting specimens under snowfall conditions to observe the snow-melting effect of the salt-storage asphalt mixture.

The test observation was conducted in Shenyang City, Liaoning Province, on December 23rd, under the weather conditions of light snow with the ambient temperature ranging from −7 °C to −4 °C during the observation. During snowfall, the surface of the ordinary asphalt mixture was gradually and evenly covered with snow, while the solution formed on the surface of the salt-storage asphalt rutting specimens. After the snowfall ceased, no snow accumulation or icing was observed on the specimen surfaces. Based on the surface conditions of the rutting plates, the ice and snow melting performance grade of the asphalt mixture incorporated with self-prepared slow-release materials was evaluated as excellent.

The ice melting rate test was used to determine the ice melting performance of the salt-storage asphalt mixture. In accordance with the existing standards, 40 g of deionized water was weighed and poured into silica gel ice cube molds, then frozen at −18 °C for 24 h for subsequent use. Meanwhile, the Marshall specimens were placed in the low-temperature chamber at −5 °C for 4 h for constant temperature conditioning. The ice cubes were quickly taken out and weighed, then placed on the surface of the Marshall specimens, which were then kept in the −5 °C low-temperature chamber for another 2 h of constant temperature incubation. After that, the ice cubes were removed and their residual mass was weighed. The ice melting rate was calculated according to Formula (6), with the results accurate to 0.1%. The test results are presented in Table 3.

(6) $$\sigma =\frac{M-m}{M}\times 100\%$$

where σ denotes the ice melting rate, M represents the initial mass of the ice cube in grams (g), and m stands for the residual mass of the ice cube in grams (g) after heat preservation at −5 °C for 2 h.

According to the data in Table 3, at the test duration of 2 h, the ice melting rate of the ordinary asphalt mixture was only 2.48%. This indicates that ice hardly melted at −5 °C, and the 2.48% ice melting rate was merely attributed to the combined effects of the test environment and sublimation. In accordance with the requirements of existing industrial standards that the ice melting rate of salinized materials shall be no less than 20%, the 5% salt-storage asphalt mixture achieved an ice melting rate of 56.35%, which meets the specified standard requirements.

#### 5.1.2. Test on Long-Term Ice and Snow Melting Performance

**Figure 10:**
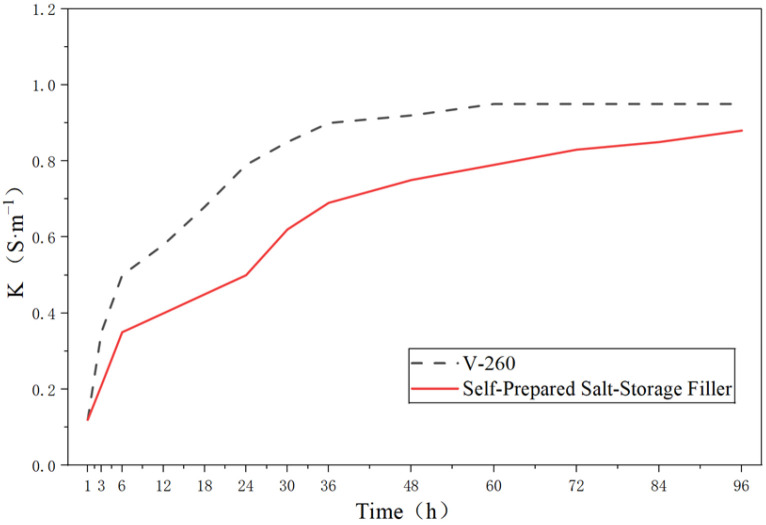
Long-term ice and snow melting performance test.

Natural immersion is the common test method, typically used to determine the salt leaching amount of materials. Molded specimens were placed in the container, a certain volume of water was poured in, and then the immersion was conducted under room temperature conditions. During the immersion of specimens in water, the salt contained therein would dissolve slowly, and the electrical conductivity of the water would also change over time. The amount of salt leached from the specimens could be inferred by testing the electrical conductivity of the water after immersion. Marshall specimens of asphalt mixture with 5% V-260 content and asphalt mixture with 5% self-prepared salt-storage filler content were prepared in accordance with the mix proportion of ordinary asphalt mixture mentioned above. The specimens were placed in a container with deionized water at room temperature (16.5 °C), and the liquid level was required to cover the top of the specimens. The electrical conductivity of the water in the container was tested at specific time intervals, and the long-term ice and snow melting performance of the specimens was evaluated based on the test results. The test results are shown in Figure 10.

**Figure 11:**
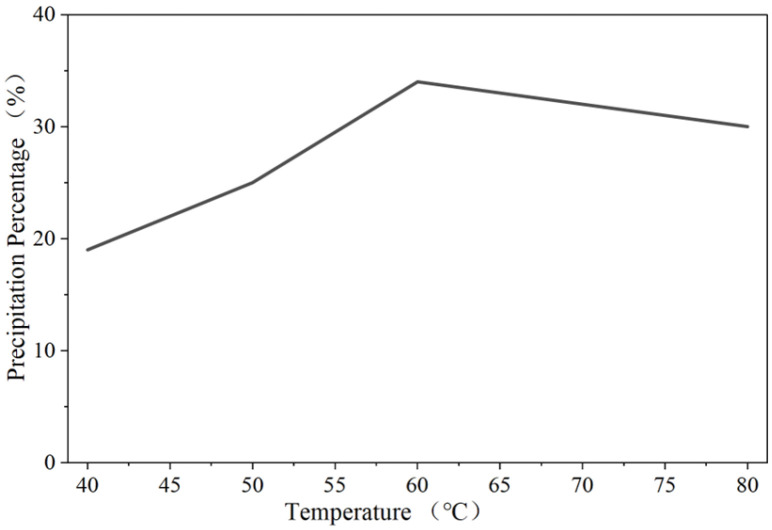
Leaching percentage of specimens at different temperatures.

From the comparison of conductivity changes between the self-prepared slow-release salt-storage filler and V-260 in Figure 11, it can be seen that V-260 reached its release limit at 60 h with almost no further change in conductivity, while the conductivity of the self-prepared salt-storage filler did not tend to be stable until 84 h of immersion. This indicates that the self-prepared slow-release salt-storage filler has better slow-release characteristics and superior long-term ice and snow melting performance, extending the effective release duration by 60%. In addition, the salt release process of the asphalt mixture with the self-prepared slow-release salt-storage filler can be divided into three stages, as shown in the figure. The first is the rapid dissolution stage from the start to 6 h. The second is the stable release stage with the constant release rate, lasting from 6 h to 72 h. The final stage is the slow dissolution stage after 72 h, which is mainly characterized by a decreased release rate.

The reason for the rapid initial salt dissolution is that the small portion of salt remained on the surface of the filler during its preparation, which dissolved quickly upon contact with water. Additionally, the simultaneous dissolution of salt inside the filler contributed to the high dissolution rate in the first stage. However, the constant salt dissolution rate in the second stage is mainly attributed to two counterbalancing effects: one is the increased release of salt from the filler due to capillary attraction, and the other is the gradual slowdown of salt desorption from the filler surface. Finally, the reduced dissolution rate in the third stage is primarily caused by the completion of salt desorption from both the surface and the interior of the filler. During this period, the ice and snow melting performance is far lower than that in the previous stages, and the effective dissolution area has been greatly reduced; thus, the salt dissolution process slows down accordingly.

### 5.2. Salt Release Characteristics Under Different Temperature Conditions

**Figure 12:**
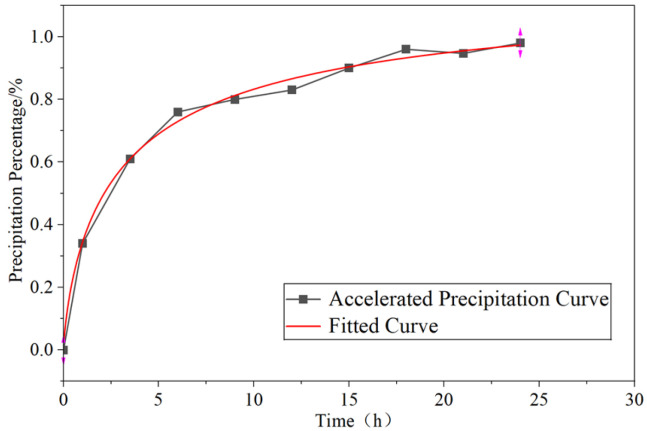
Accelerated immersion test.

Self-prepared salt-storage fillers were incorporated into the asphalt mixture at a dosage of 5%, and the ion leaching rate was measured after immersion for 48 h at 40 °C, 50 °C, 60 °C, 70 °C, and 80 °C, as shown in Figure 12. The leaching percentage was calculated according to Formulas (7)–(9), and the optimal temperature was determined, with the test results presented in Figure 12. It should be emphasized that the 60 °C immersion test is designed as an accelerated aging protocol, not as a simulation of actual pavement temperature. Field pavements in cold regions rarely experience bulk water immersion at 60 °C; rather, this elevated temperature serves to magnify the dissolution and diffusion kinetics of the encapsulated salts within a controlled laboratory timeframe.

(7) $$m_{1}=\frac{M}{\left(1+OAC\right)}\times r$$

(8) $$A=m_{1}\times c$$

(9) $$P=m_{2}(\rho_{2}-\rho_{1})/(A\times \rho_{1})$$

where M denotes the mass of the specimen in grams (g), r represents the ratio of the amount of salt-storage filler added to the aggregate in percentage (%), m_1_ stands for the amount of salt-storage filler added in the specimen in grams (g), OAC is the optimal asphalt-aggregate ratio in percentage (%), ρ_1_ denotes the specific gravity of water at the fixed temperature in grams per milliliter (g/mL), ρ_2_ represents the specific gravity of the immersion solution in grams per milliliter (g/mL), A stands for the content of effective components in the salt-storage filler within the specimen in grams (g), P denotes the percentage of precipitated effective components in percentage (%), m_2_ represents the amount of solvent in grams (g), and c stands for the content of chloride-based salts in percentage (%).

The salt leaching percentage of the asphalt mixture increases gradually with the rise in temperature, peaking when the temperature reaches 60 °C. This is because the thermal motion of molecules intensifies as the temperature rises, which increases the solubility and thus accelerates the rate of salt leaching from the mixture. However, the leaching percentage begins to decline with a further increase in temperature. The reason for this is that when the temperature exceeds 60 °C, the water permeability of the asphalt mixture is enhanced and the permeability of the mixture is no longer affected by temperature, resulting in no further acceleration of salt leaching. Meanwhile, the rising water temperature causes the asphalt to gradually soften and leach out; the hot-melt colloid structure becomes loose, the intermolecular forces of the hot-melt colloid are weakened, and the colloidal molecules lead to the blockage of some pores. This further slows down the rate of salt leaching and may prevent more salt from leaching out of the mixture, ultimately resulting in a decrease in the salt leaching percentage.

**Figure 13:**
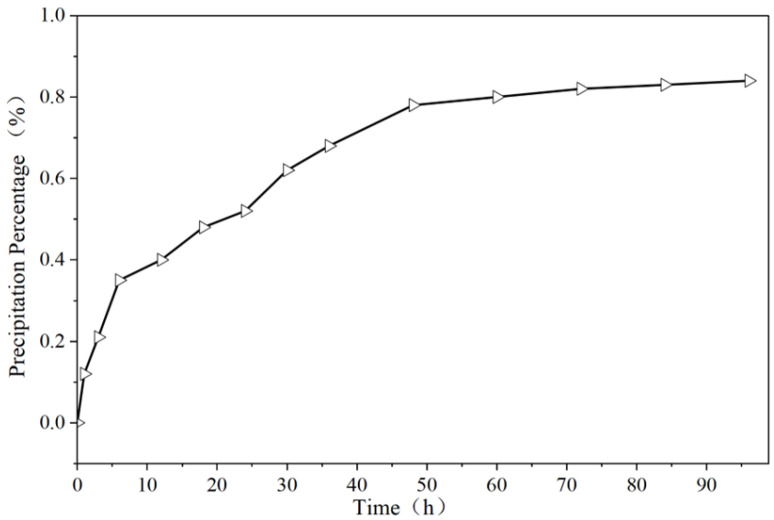
Natural immersion test.

**Figure 14:**
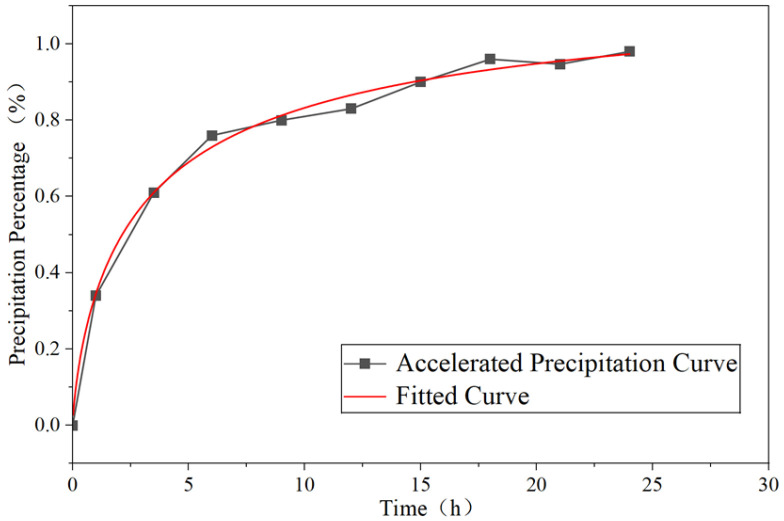
Extended comparison of accelerated immersion tests.

Therefore, in this experiment, 60 °C was selected as the temperature for the accelerated immersion test. This ensures the maximization of the salt leaching rate while avoiding adverse effects such as denaturation of the asphalt mixture and pore blockage caused by excessively high temperatures. The accelerated dissolution test was conducted at 60 °C, and logarithmic fitting was performed on the test data to show the relationship between the dissolution percentage and time, which was then compared with the natural leaching percentage curve, as shown in Figure 13 and Figure 14. The logarithmic fitting Formula (10) can be expressed as follows.

(10) $$P_{1}=0.1939ln(t_{1})+0.3619(t_{1}\geq 0.25)\;(R^{2}=0.9552)$$

where P_1_ denotes the salt release percentage (unit: %), t_1_ represents the salt release time with the minimum value of 1 h (unit: h), and R_2_ stands for the correlation coefficient.

To predict the functional service life of the salt-storage asphalt pavement, a three-step methodology was established. First, the accelerated immersion test was conducted at 60 °C to obtain the salt-release curve under elevated-temperature conditions. Second, a logarithmic fitting was performed on the accelerated test data to derive the quantitative relationship between salt-release percentage and immersion time (Equation (10)). Third, considering that the salt leaching amount in the accelerated test was approximately six times that observed in the natural immersion test, the time axis of the accelerated curve was extended by a factor of six to simulate the natural salt-release process. The extended curve was then compared with the natural leaching curve to verify its reliability. Once validated, the extended dissolution data were converted from hours to years, and two correction coefficients—the leaching area conversion coefficient (*K*) and the immersion conversion coefficient (*W*)—were introduced to account for the differences between laboratory specimens and actual pavement field conditions. The final predictive model is expressed in Equation (12).

A comparison of Figure 12 and Figure 13 reveals that the dissolution trend of the asphalt mixture in the accelerated immersion test is similar to that in the natural immersion test, with both showing the gradual decrease as time elapses. In addition, the test results also indicate that the salt leaching pattern in the accelerated immersion test follows the same three-stage leaching characteristic as observed in the natural one. These findings verify that the accelerated immersion test can replace the natural immersion test, as it can effectively simulate the natural immersion process and accurately reflect the dissolution behavior of asphalt mixtures in snow-melting environments, thus enabling the evaluation of the long-term snow-melting performance of salt-storage asphalt mixtures.

### 5.3. Prediction of Functional Service Life

The characteristic of salt-storage asphalt mixture to actively leach salt plays a crucial role in melting ice and snow on pavement in snowy weather. The more salt leached out, the better the snow-melting performance. Meanwhile, the duration of salt leaching is also an important reflection of the ice and snow-melting capacity of salt-storage asphalt mixture. With the passage of time, under conditions such as high temperature and heavy rainfall in summer and wheel abrasion, salt in the salt-storage pavement will be continuously lost, and its long-term ice and snow-melting capacity will decline steadily. This also results in the shortened functional service life of the salt-storage pavement, which is inconsistent with its service life.

In this study, the long-term salt leaching percentage was adopted to evaluate the long-term snow-melting performance of the mixture. Given that the dissolution trend in the accelerated test was similar to that in the natural dissolution test, a more reasonable salt leaching pattern could be derived based on the results of both natural and accelerated leaching tests. Since the amount of salt leached in the accelerated test was approximately six times that in the natural leaching test, the new set of data could be obtained by multiplying the test duration of the accelerated test by six. Finally, the new curve was plotted based on the extended dissolution data. Figure 14 shows the curve and its logarithmic fit. The logarithmic fit for the extended dissolution data can be expressed as follows.

(11) $$\begin{array}{l} P_{2}=0.1939ln(t_{2})+0.3619\;(t_{2}\geq 1)\;(R^{2}=0.9147) \end{array}$$

where P_2_ refers to the salt release percentage in percentage (%), t_2_ denotes the salt release time with the minimum value of 1 h (h), and R_2_ represents the correlation coefficient.

As shown in Figure 14, the logarithmic fit provides an excellent match to the test results with a fairly high goodness of fit (R^2^ = 0.9147). Additionally, the trend of the curve for the extended accelerated immersion test data is consistent with that of the natural leaching curve, which verifies that Formula (12) can be used to evaluate the long-term ice and snow-melting performance of salt-storage asphalt mixtures.

In the fitting Formula (11), the unit of t_2_ is hours (h). Converting it to years based on 365 days and multiplying by the correction coefficient, the model for predicting the service life is derived, with the calculation formula shown in Formula (12).

(12) $$T=\frac{e^{\left(\frac{P_{2}\;+\;0.3619}{0.1939}\right)}\times K\times W}{8760}$$

where T denotes the predicted service life in years, P_2_ represents the salt release percentage in %, K is the conversion coefficient of salt release area, and W stands for the immersion conversion coefficient.

In the formula, S_1_ denotes the salt release area with the unit of square meters (m^2^), r represents the radius of the asphalt Marshall specimen in millimeters (mm), and h stands for the height of the asphalt Marshall specimen in millimeters (mm).

Only the upper surface of an actual salt-storage asphalt pavement is in contact with ice and snow, so salt leaching occurs solely on the upper surface. The corresponding area calculation formula is shown as Formula (13) below.

(13) $$S_{2}=\pi r^{2}\times 10^{-6}$$

It can thus be concluded that the leaching area conversion coefficient is K = S_1_/S_2_ = 3.5. According to the statistics from Liaoning Meteorological Bureau, the annual average rainfall from 2020 to 2024 was 586.1 mm, 687.2 mm, 710.0 mm, 888.0 mm, and 892.0 mm, respectively, with the average annual rainfall over the recent five years reaching 752.66 mm. The water consumption in the immersion test was 7 L, giving an immersion conversion coefficient of W = 9.30. Substituting these values into Formula (12), it is obtained that the service life of the slow-release salt-storage asphalt pavement with ice and snow-melting performance in Liaoning is 4.07 years.

However, the prediction model presented in Equation (12) requires adaptation for different conditions. For other mixture gradations, accelerated dissolution tests can be conducted to establish analogous predictive equations. Temperature and precipitation are critical environmental parameters influencing the long-term snow-melting performance of salt-storage asphalt pavements, while traffic axle load represents another significant factor that cannot be overlooked. Furthermore, the salt precipitation rate in accelerated immersion tests exhibits equivalence to the dissolution behavior under natural conditions. The transformation from laboratory conditions to field environments can be achieved through the immersion conversion coefficient (W = 9.30) and the precipitation area conversion coefficient (K = 3.5).

It should be noted that this prediction model does not fully account for the combined effects of complex environmental factors on salt loss, including traffic-induced abrasion, freeze–thaw cycles, and temperature fluctuations. Moreover, future climate change may cause actual service conditions to deviate from historical trends. These simplifying assumptions may introduce certain uncertainties; therefore, the predicted value of 4.07 years should be interpreted as a theoretical estimate based on existing accelerated test conditions and specific climatic parameters, rather than an exact in-service lifespan. It is recommended that subsequent research validate and refine the reliability of this prediction model through long-term field exposure tests. Although the factors affecting snow-melting performance, such as snowfall amount, ambient temperature, moisture content, and axle load, are more complex than those in this study, the present investigation provides a valuable contribution to the understanding of long-term salt-release patterns in salt-storage asphalt pavements.

## 6. Conclusions and Recommendations

In this paper, the carrier, snow-melting salt, water repellent, polymer coating material, preparation method, and other components were selected to prepare the slow-release salt-storage filler, and the road performance of the slow-release salt-storage filler was evaluated. The following conclusions were obtained:

(1) From the freezing point depression tests of four types of soluble salts, including KCl, it can be concluded that calcium chloride (CaCl_2_) and sodium chloride (NaCl) exhibit strong freezing point depression capacity. According to the dissolution heat data, CaCl_2_ and NaCl are suitable for being formulated into snow-melting salt. To reduce costs, the compound ratio of CaCl_2_ to NaCl was determined to be 2:1 through conductivity and ice-melting compounding tests. Finally, BET specific surface area measurements were carried out on five types of carriers, such as mineral powder with different particle sizes. Attapulgite was selected as the optimal carrier, and the salt-storage filler was prepared by the wet adsorption method.

(2) Based on the tests in this paper, the optimal mix proportion and the best asphalt-aggregate ratio were confirmed to be 4.92%. The self-prepared slow-release salt-storage filler was added by the external mixing method, and the dosage should not exceed 5% to ensure pavement performance. The addition of salt-storage filler has a negative impact on the high-temperature deformation resistance and water damage resistance of asphalt mixtures. With the increase in dosage, the high-temperature deformation resistance decreases by 6.4–42.6%, and the water damage resistance decreases by 2.5–13.3%. The addition of salt-storage filler causes the low-temperature crack resistance of asphalt mixtures to first increase and then decrease, reaching the peak when the dosage is 2%, with an increase of 12.2%. Among these properties, the salt-storage filler has an extremely significant impact on the water stability of asphalt mixtures at the same dosage. The influence of salt-storage materials on the pavement performance of asphalt mixtures is mainly reflected in increasing the porosity of mixtures, reducing the adhesion of asphalt mortar after salt dissolution and segregation, and changing the structural ratio of asphalt mortar.

(3) The self-prepared salt-storage filler can achieve a remarkable ice and snow-melting effect when incorporated into asphalt mixtures, and it exhibits excellent slow-release performance after coating. The long-term effectiveness of the self-prepared slow-release salt-storage filler is superior to that of V-260, with the slow-release duration extended by 60%. However, the salt-storage capacity of the self-prepared salt-storage filler is lower than that of V-260, showing the 11.8% reduction. Immersion tests were conducted on the salt-storage asphalt mixtures under different temperature conditions. By determining the leaching percentage at various temperatures, it was confirmed that the leaching percentage reached the maximum at 60 °C, and this temperature was thus selected as the accelerated immersion temperature. A comparison of the leaching percentage results from the accelerated immersion test and the natural immersion test revealed that the salt leaching patterns were essentially consistent under different temperature conditions. Meanwhile, logarithmic fitting was performed on the results of the accelerated immersion test, followed by data extension; the comparison of the extended test results with those of the natural immersion test showed that the extended results were approximately consistent with the natural leaching results.

Three distinct implementation strategies are proposed for incorporating slow-release salt-storage fillers into asphalt pavements: (1) surface infiltration, whereby suspensions are applied through pavement voids or micro-cracks and capillary action transports salts into the near-surface asphalt mastic; (2) drilled injection, involving controlled boreholes (10–20 mm in diameter, 30–50 mm in depth) in the wearing course, followed by pressure injection of filler slurry and sealing with high-performance patching materials; and (3) mill-and-replace, entailing partial milling of the wearing course (20–40 mm), blending fresh filler with new asphalt mixture, and overlaying the existing structure. These methods offer differentiated technical advantages suited to diverse pavement conditions, significantly broadening engineering applicability: surface infiltration is appropriate for pavements with minor surface deterioration, drilled injection enables targeted, minimally invasive localized treatment, and mill-and-replace is recommended for more severe surface distress requiring comprehensive material integration. Collectively, these approaches establish a robust methodological foundation for subsequent research on sustainable salt-storage pavement maintenance, encompassing long-term deicing performance evaluation, optimization of salt-release kinetics, and multi-scale mechanical compatibility assessment, thereby advancing the development of environmentally friendly and durable winter road maintenance technologies.

## Figures and Tables

**Table 1:** Marshall test results of AC-13 asphalt mixture.

Oil-Stone Ratio (%)	Relative Bulk Density	Theoretical Maximum Density	Void Percentage (%)	Voids in Mineral Aggregate (%)	Asphalt Saturation (%)	Stability (kN)	Flow Value (mm)
4.0	2.406	2.554	5.8	13.8	57.9	10.49	2.40
4.5	2.411	2.535	4.9	14.0	65.1	11.51	2.64
5.0	2.417	2.517	4.0	14.2	72.0	11.40	2.72
5.5	2.425	2.499	3.0	14.3	79.3	10.68	2.84
6.0	2.426	2.482	2.3	14.7	84.6	9.30	3.58
design requirements	—	—	3~5	>14	65~75	>8	2~4

**Table 2:** Specific surface area of powders with different particle sizes.

Sample	Specific Surface Area (m^2^·g^−1^)	Cumulative Desorption Area (m^2^·g^−1^)	Cumulative Desorption Volume (cm^3^·g^−1^)	Cumulative Desorption Diameter (4A·V^−1^)
Attapulgite	226.1	37.81	0.1	92.14
Fly Ash	8.597	4.11	0.01	41.25
Diatomite	116.324	18.89	0.08	80.29
Volcanic Rock	98.067	11.78	0.02	49.26
Calcium Carbonate	3.981	3.02	0.006	39.78

**Table 3:** Test results of ice melting rate.

Specimen Type	Test Results		
	M/g	m/g	*σ*/%
5% Salt-Storage Asphalt Mixture	39.4	17.2	56.35
Ordinary AC-13 Asphalt Mixture	40.3	39.3	2.48

## Data Availability

The original contributions presented in this study are included in the article. Further inquiries can be directed to the corresponding author.
